# Ultra-thin Graphitic Film: Synthesis and Physical Properties

**DOI:** 10.1186/s11671-016-1283-2

**Published:** 2016-02-01

**Authors:** Tommi Kaplas, Polina Kuzhir

**Affiliations:** Institute of Photonics, University of Eastern Finland, Yliopistokatu 7, 80101 Joensuu, Finland; Research Institute for Nuclear Problems, 11 Bobrujskaya Str., Minsk, 220030 Belarus; Ryazan State Radio Engineering University, 59/1 Gagarina Street, Ryazan, 390005 Russia

**Keywords:** Graphene, Pyrolytic carbon, Thin film, CVD synthesis, Transfer process, Optical properties, Sheet resistance

## Abstract

A scalable technique of chemical vapor deposition (CVD) growth of ultra-thin graphitic film is proposed. Ultra-thin graphitic films grown by a one-step CVD process on catalytic copper substrate have higher crystallinity than pyrolytic carbon grown on a non-catalytic surface and appear to be more robust than a graphene monolayer. The obtained graphitic material, not thicker than 8 nm, survives during the transfer process from a Cu substrate without a template polymer layer, typically used in the graphene transfer process to protect graphene. This makes the transfer process much more simple and cost-effective. Having electrical and optical properties compatible with what was observed for a few layers of CVD graphene, the proposed ultra-thin graphitic film offers new avenues for implementing 2D materials in real-world devices.

## Background

Graphene, one monolayer of graphite, is one of the most multifunctional materials. Many intrinsic properties of graphene, including high mechanical strength, flexibility, transparency, and good electrical conductivity, make it appealing for a wide variety of applications [1]. These applications could be found, e.g., in medicine [2], ultra-fast electronics [3], energy harvesting [4], and telecommunications [5].

Synthesis of graphene by an inexpensive and simple technique, enabling the production thereof up to meter scale, is one of the most important breakthroughs towards the graphene industrial revolution [6, 7]. Catalyst-assisted chemical vapor deposition (CVD) [6, 8, 9] is the most promising candidate to develop a cost-effective scalable protocol of high-quality graphene manufacturing as compared to other well-known methods of producing graphene, such as mechanical exfoliation of graphite [10] and sublimation of epitaxial SiC [11].

Despite CVD technique is recognized as one of the top candidates for graphene mass production, the drawback of CVD graphene is the transfer stage: in the CVD process, graphene is deposited on a transient metal, such as nickel or copper, and thereafter transferred on a final substrate typically through the spin-coating technique. Although the covalent C–C bonds ensure remarkable mechanical properties to graphene monolayer [12–14], each phase of transferring, i.e., spin-coating with polymetilmetacrilate (PMMA), heating, etching of the remaining metal, and transferring to the substrate for final usage, may damage initially high-grade graphene. This is the reason why the graphene portfolio could suffer in benchmarking with other mechanically robust electrically conductive transparent thin films, such as ITO.

In this sense, for many applications it could be sufficient to use slightly thicker carbon-based material instead of a graphene monolayer. A multilayered graphene could be a good candidate, but it is noteworthy that synthesizing homogeneous multilayered graphene with a desired number of layers is not a trivial task.

Pyrolytic carbon or pyrocarbon (PyC) is a disordered carbon material which has been used as thin films for decades [15, 16]. It is made of nanoscale graphitic domains connected by domain boundaries, containing defects such as vacancies and dislocations, and displays turbostratism [17]. The synthesis by CVD of few nanometer-thick pyrocarbon layers from hydrocarbon precursors was for the first time reported in ref. [16]. More recent contributions of refs. [18, 19] differ from [16] by the nature of the substrate and the absence of a posterior graphitization step. Typically, these sub-100-nm-thick films are almost atomically smooth. According to our previous investigations, PyC being transparent [20, 21], conductive [18, 22], and at the same time robust enough to survive in a harsh environment [23] could compete with multilayered graphene in many aspects. But in contrast to graphene, PyC can be grown via CVD at any substrate, either metal or dielectric, that can sustain at high processing temperature conditions (1000 °C).

In this letter, we demonstrate a one-step technique to grow a polycrystalline, graphitic thin film material on a copper substrate. The resultant sub-10-nm-thick graphitic pyrocarbon (GrPyC) film has been investigated using Raman spectroscopy, optical and SEM microscopy, and dc conductivity measurements and has been compared with graphene.

## Methods

The GrPyC film was grown on a Cu foil (99.8 % pure) using the CVD setup described in details elsewhere [18]. The Cu foil was first heated to 1000 °C in hydrogen atmosphere (constant flow 0.1 mBar), and then the CVD chamber was filled with methane (static atmosphere 25 mBar). The graphitization process lasted 30 min in total, after which the chamber was pumped to vacuum and cooled in a static hydrogen atmosphere (5 mBar, overnight cooling).

**Fig. 1:**
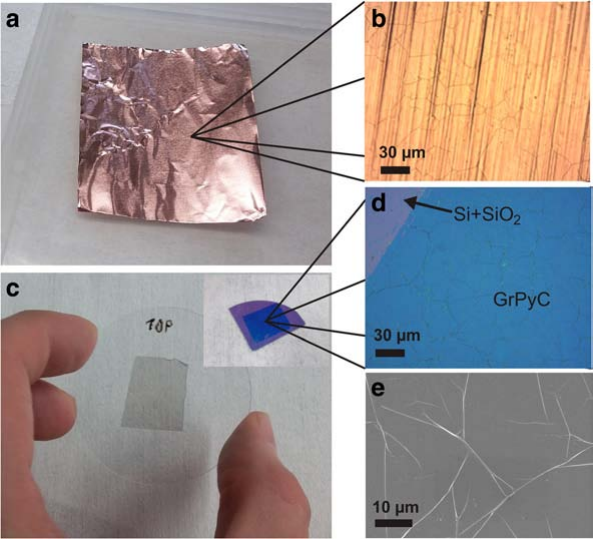
**a**, **b** Copper foil, used as a substrate after the CVD, looks very similar to that of the original copper foil. **c** GrPyC transferred on a silica substrate and on Si/SiO2 (300 nm)—*inset*. **d**, **e** A closer look with an optical microscope and scanning electron microscope shows that although there are wrinkles all around the sample, there are no color nor contrast differences which indicate very uniform film thickness

The ultra-thin graphitic pyrocarbon film was transferred from copper to a silica substrate and on silicon coated with thermal SiO_2_ (300 nm). For optical and electric measurements, transferring was done using a conventional routine procedure as for monolayer graphene with polymer support [24]. At first, the GrPyC/Cu sample was coated with PMMA (500 nm), and then the backside GrPyC was etched away with harsh oxygen plasma (100 W/20 sccm/2 min). After reactive ion etching, Cu was etched away with a FeCl_3_ solution and PMMA/GrPyC was rinsed in pure water for 1 hour twice. Next, the sample was deposited on a dielectric substrate, and the PMMA layer was removed by acetone. In Fig. 1, one can see the original Cu film coated with GrPyC and the carbon film transferred on dielectric substrates.

The thickness of the GrPyC film was measured by using a Veeco Instruments, Dektak 150 stylus profiler. The transmittance of the GrPyC film was measured by PerkinElmer lambda-18 over a spectral range of 230–800 nm and an empty silica substrate as a reference. The Raman spectrum was measured by inVia Raman microscope using 514-nm excitation wavelength with low excitation power in order to avoid heat-induced effects. Scanning electron microscopy imaging was done with SEM LEO 1550 Gemini. The sheet resistivity was measured using a four-point probe technique with 3-mm probe spacing.

## Results and Discussion

**Fig. 2:**
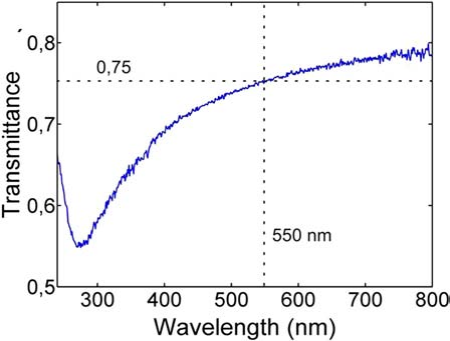
Optical transmittance of GrPyC

The optical transmittance spectrum in Fig. 2 resembles that measured on graphite rather than that found on PyC having the absorption peak maxima at 270 nm [25]. At the midpoint of visible spectral range, 550 nm wavelength, the transmittance is about 75 %. If the reflectance of the film is approximated close to zero, the absorption of the ultra-thin graphitic film would be more than 20 % of the incident light. Since the thickness of a graphene monolayer, absorbing 2.3 % of light, is 0.34 nm [26], the collected optical data for GrPyC suggest that the film consists of about ten graphene layers. In this case, its thickness should be approximately 3.4 nm. However, the stylus profiler measurements showed that GrPyC is 8-nm ± 1-nm thick, which is more than twice the estimated value.

**Fig. 3:**
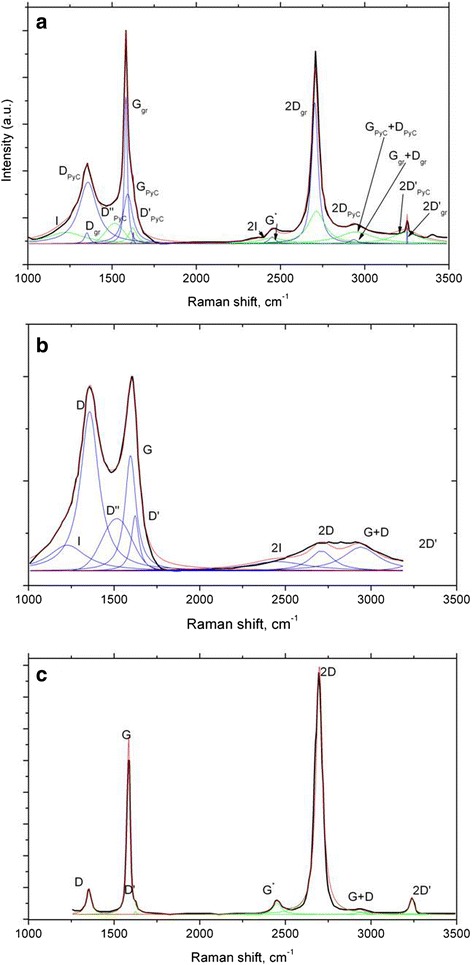
**a** Raman spectrum of GrPyC and source spectrum of **b** PyC and **c** CVD-grown defected graphene monolayer

The Raman spectrum of GrPyC film in Fig. 3a shows that the G peak at 1582 cm^−1^ is very narrow (full-width-half-maximum is ~40 cm^−1^) and resembles to that of measured from graphite rather than that of found from PyC, where the width is typically more than 100 cm^−1^ [27, 28]. Such a narrow and strong G mode indicates the presence of highly crystalline graphitic material.

At 2710 cm^−1^, one can observe a strong 2D peak. In the amorphous PyC, the 2D peak is not found, but it is usually related to a more crystalline graphitic carbon. However, on the right-hand side, one can observe small shoulder in 2D (at 2780 cm^−1^). This wide peak is typical for Raman spectra of PyC, which indicates the presence of an amorphous PyC-like material [18].

The presence of D peak at 1350 cm^−1^ is related to the disordering in the graphitic lattice. D mode is strong having intensity of about half in comparison to the G peak. This shows that the carbon film not only consists of highly crystalline graphitic structure but also contains more defective sp^2^ carbon, i.e., some pairs of 5- and 7-membered rings exist in the material to give a contribution to the D band. Moreover, the D´ mode at 1550 cm^−1^ indicates that the film contains not only disordered sp^2^ carbon but also small amounts of sp^3^ hydrogenated carbon [27, 28].

In Fig. 3b, c, typical Raman spectra of PyC and a defected graphene monolayer produced by CVD technique are presented, respectively. Comparing the dominating peaks (see Fig. 3a–c), one can notice that all the peculiarities of GrPyC spectrum (Fig. 3a) are inherent to graphene and PyC as well.

The measured sheet resistance was 2.08 kΩ^−1^ for a monolayer graphene and 1.15 kΩ^−1^ for GrPyC film. Although, ultra-thin graphitic film is more than 20 times thicker than a single-layer graphene, the sheet resistance of the film is only about half of that of graphene. This can be explained by the amorphous fraction of the GrPyC material, which is supposed to be less conducting in comparison with graphite due to a strong electron scattering.

A graphene monolayer is conventionally grown on a copper substrate with low hydrocarbon concentration. Because of the low carbon solubility, and of the limited amount of reactant hydrocarbons, the CVD process typically results only in a monolayer graphene [29], which covers completely the Cu surface. However, the increased amount of hydrocarbon involved in the process will lead to the formation of a thicker carbon layer as shown in this paper.

The mechanism behind this is the following: first, a graphene monolayer grows on the copper substrate, taking benefit from the catalytic effect to achieve a very high crystalline order. Then, deposition continues over this first layer, but without the catalytic effect of copper, which is now masked by the first graphene layer; consequently, more defects are introduced and the crystalline size of the graphenic domains diminishes, as reported in “templated growth” experiments [30].

The quality of a transparent conductive film can be estimated by the electrical to optical conductivity ratio [29]. The calculated ratio for GrPyC (~1 at 550 nm) is lower than for single-layer graphene (~7 at 550 nm). However, this value is greater for GrPyC than for a PyC film of the same thickness (~0.4 at 550 nm for an 8-nm-thick PyC) [18, 31].

Despite the fact that GrPyC does not have as high a quality factor as graphene does, there is a significant benefit, which gives GrPyC an advantage over graphene. A graphene monolayer without an external polymer layer does not survive during the transfer process. This will hamper the use of graphene because the polymer often leaves some remains on the graphene surface after the transfer. Moreover, in an industrial scale, the extra polymer layer increases the cost of the resultant material.

**Fig. 4:**
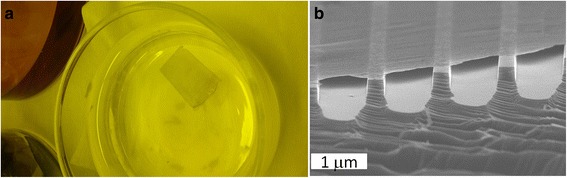
**a** About 1 cm × 2 cm GrPyC film transferred from a Cu substrate to water. **b** GrPyC film deposited on a grating structure. Transfer processes here were done without supporting PMMA layer

In our experiment, we were able to transfer a 1-cm scale GrPyC layer without spin-coated polymer; see Fig. 4a: the GrPyC film was deposited on a Si grating without using a PMMA protective layer. In Fig. 4b, we can observe how the GrPyC film remains on the top of the grating structure. This indicates that the GrPyC material could be used, e.g., as a membrane for micro- and nanoelectromechanical systems. Because of its strength, the deposition of a GrPyC film could be easier on micro- and nanostructures in comparison to a single-layer graphene.

## Conclusions

In summary, the CVD-grown nanometrically thin graphitic film on a copper substrate was observed to consist of graphitic carbon with higher crystallinity than amorphous PyC. The sheet resistance of the GrPyC film was lower than that of graphene, while still preserving a rather high transparency. The obtained sub-10-nm-thick GrPyC film demonstrates outstanding robustness: the film survives during the transfer process from a Cu substrate on a microstructure without a template polymer layer. We believe that a significant reinforcement effect of PyC on graphene multi-layers offers new avenues for implementing graphene and graphene-like materials in real-world devices.

## Abbreviations

CVD: chemical vapor deposition
GrPyC: graphitic pyrocarbon
PMMA: polymetilmetacrilate
PyC: pyrolytic carbon or pyrocarbon
SEM: scanning electron microscopy
